# Transparent and Privacy-Preserving Mobile Crowd-Sensing System with Truth Discovery

**DOI:** 10.3390/s25072294

**Published:** 2025-04-04

**Authors:** Ruijuan Jia, Juan Ma, Ziyin You, Mingyue Zhang

**Affiliations:** College of Computer and Information Science College of Software, Southwest University, Chongqing 400010, China; ruijuanjia1@gmail.com (R.J.); mj321000311@email.swu.edu.cn (J.M.); youziyin1999@gmail.com (Z.Y.)

**Keywords:** mobile crowd sensing, truth discovery, transparency, privacy-preserving, zero-knowledge proof

## Abstract

The proliferation of numerous portable mobile devices has made mobile crowd-sensing (MCS) systems a promising new trend. Traditional MCS systems typically outsource sensing tasks to the data aggregator (e.g., cloud server). They collect and analyze the provided sensing data through an appropriate truth discovery (TD) method to identify valuable data sets. However, existing privacy-preserving MCS systems lack transparency, enabling data aggregators to deviate from the specified protocols and allowing malicious users to provide false or invalid sensing data, thereby contaminating the resulting data sets. The lack of transparency and public verifiability in MCS systems undermines widespread adoption by preventing data requesters from confidently verifying data integrity and accuracy. To address this issue, we propose a transparent and privacy-preserving mobile crowd-sensing system with truth discovery (TP-MCS) constructed using zero-knowledge proof (ZKP) and the Merkle commitment tree. This scheme enables data requesters to effectively verify the correctness of the truth discovery service while ensuring data privacy. Furthermore, theoretical analysis and extensive experiments demonstrate that this scheme is secure and efficient.

## 1. Introduction

With the rapid development of mobile devices and wireless technologies, the scale of data and computational complexity are increasing [1]. In this context, the widespread application of various mobile intelligent terminal devices and sensing technologies in numerous applications has promoted mobile crowd sensing (MCS) to become a popular data sensing and aggregation paradigm, fully leveraging collective intelligence. MCS systems have established a relatively mature framework in various domains, e.g., urban sensing [2], healthcare [3,4], and environmental monitoring [5]. Nevertheless, sensor quality, the surrounding environmental noise, and device mobility inevitably lead to differences and even contradictions between sensing data. As a new sensing paradigm, crowd sensing typically relies on a centralized crowd-sensing platform [6,7], which uses truth discovery to distribute sensing tasks to individuals or groups for collecting and analyzing sensing data from mobile devices.

In practice, when sensing users provide personal sensing data, there is a risk of privacy leakage. If the reliable information users provide is compromised and leaked, the service provider (SP) may abuse it, resulting in negative consequences and potential dangers for participating users [8]. It may even lead to user resistance to participating in sensing tasks. If users lack enthusiasm for participating in sensing tasks, the MCS system may fail to provide satisfactory data and service. Therefore, maintaining the privacy of users’ sensing data is of paramount importance. Existing research addresses privacy issues using methods such as homomorphic Paillier encryption [9], Shamir’s secret sharing technology [10], or the introduction of two non-colluding cloud servers [11]. They ensure secure data transmission by strictly encrypting the sensing data or weights during interaction. However, these existing schemes overlook the risk of malicious users submitting anomalous sensing data.

We notice malicious users injecting random or invalid data within this task-outsourcing framework for unforeseen reasons. Undoubtedly, such behavior not only increases the workload on the server side but also causes aggregation pollution, affecting system performance and computational accuracy, leading to a deviation of the final data results from the truth. Current research has employed the blockchain-driven zero-knowledge range proof (ZKRP) technique [12] and an authentication-based approach [13] to improve the reliability of results derived from mixed sensing data. These methods aim to prevent anomalous data values or fake users. Meanwhile, the work [13] could not detect abnormal data from legitimate users. In addition, these schemes are executed by the cloud server, rendering them unable to defend against tampering with aggregation results by the cloud server. The centralized architecture lacks operational transparency during the aggregation process [14,15], leading to the trust authority (TA) being unable to verify the complete algorithm execution process and refusing to receive incorrect aggregation results, thus reducing the credibility of MCS systems and preventing widespread adoption by cautious TA. Therefore, it is an urgent problem to realize verification in existing MCS systems.

To address the above problem, we propose a transparent and privacy-preserving mobile crowd-sensing system with truth discovery (TP-MCS). The scheme is based on zero-knowledge proof, ensuring that TA can efficiently verify the correctness of the truth discovery service while safeguarding data privacy. Meanwhile, we utilize the Merkle commitment tree data structure to optimize efficiency, which makes the transmission size of data logarithmic. In addition, we analyze the scheme’s security properties, including privacy and transparency. Finally, we describe the performance analysis and implement our scheme, and the results show that our scheme performed well.

**Contribution.** We summarize our main contributions below:

- We propose an efficient dual-verification protocol based on ZKRP and the Merkle commitment tree. On the one hand, clients can independently verify whether their data are correctly included in the algorithmic execution through Merkle commitment tree paths, thereby ensuring data authenticity and participation. On the other hand, TA achieves batch filtering of anomalous data by verifying the Merkle commitment tree root node, guaranteeing that aggregation results exclusively originate from valid data provided by honest clients. This protocol effectively ensures the integrity and tamper-resistance of the data aggregation process, consequently enhancing the system’s robustness against outliers.
- We propose a transparent and privacy-preserving mobile crowd-sensing system with truth discovery (TP-MCS). By innovatively restructuring the truth discovery framework and introducing the Merkle commitment tree range proof protocol (MTRP) and the inner-product argument weighted aggregation protocol (IAWAP) based on zero-knowledge range proof and zero-knowledge inner-product argument, our system enables comprehensive supervision and verification throughout the truth discovery process in MCS system. This approach maintains data privacy while achieving computational transparency and verifiability.
- We conduct targeted security analysis addressing potential threat models in the MCS system, demonstrating that our proposed scheme’s security properties can effectively counter various security challenges. Furthermore, we simulate realistic MCS environments to implement our solution and conduct comprehensive evaluations across multiple dimensions, including accuracy, convergence, security properties, and performance overhead. The results conclusively show that our approach maintains original accuracy and convergence characteristics while providing robust defense capabilities. Through the combination of theoretical analysis and experimental validation, we fully substantiate the solution’s practicality and scalability in real-world scenarios.

**Organization.** The remaining sections of this paper are organized as follows: In Section 2, we describe the related work of the MCS system. Section 3 provides an overview of the preliminary preparations. In Section 4, we describe the problem formulation, including the system model, threat model, and security requirements. Section 5 details the workflow of the proposed transparent and privacy-preserving mobile crowd-sensing mechanism. Section 6 describes the security analysis and communication overhead of TP-MCS. Experimental evaluations are reported in Section 7, and the discussion and limitations are described in Section 8. Finally, the conclusions are summarized in Section 9.

## 2. Related Work

In this section, we will provide a comprehensive overview of the research progress of MCS systems and explicitly state the inheritance and innovation of this selected research with the existing research results.

It is worth noting that not all data collected by users are valid. In fact, due to the diversity of data sources, there are significant differences in the quality of different data sources. If the raw data are directly used for processing, the final aggregated results will likely deviate significantly from the truth. Before the advent of the truth discovery method, MCS systems typically employed traditional approaches such as voting mechanisms or averaging calculations to resolve data conflicts [16,17]. However, these methods exhibit notable limitations, failing to account for the quality disparities among data sources adequately. Instead, they treat all data uniformly without differentiation, which significantly compromises the accuracy of the results. To address this challenge, the MCS system introduces a truth discovery scheme, which aims to improve the result accuracy through a more refined data processing mechanism. From the perspective of system design, the existing schemes mainly focus on the dimensions of privacy preservation and verifiability, which we explain next.

**Privacy-preserving MCS scheme.** In 2014, Li et al. [18] proposed a general framework called CRH, capable of resolving data conflicts and discovering truths, which could handle both discrete and continuous data. However, this framework did not take into account the protection of user data privacy. To address privacy problems in the truth discovery process, some researchers have developed solutions based on cryptographic techniques or perturbation-based methods to ensure the privacy protection of raw data. In 2015, Miao et al. [9] were the first to highlight the necessity of incorporating privacy protection into truth discovery schemes. They proposed the first privacy-preserving truth discovery protocol for MCS. The core idea of this scheme is the use of the threshold Paillier cryptosystem to securely aggregate users’ encrypted data, thereby safeguarding users’ sensitive information. However, the secure summation protocol requires frequent participation of users and servers in data encryption and decryption, imposing significant computational overhead on both entities. More critically, the scheme risks exposing the privacy of truth values during the iterative truth discovery process. To reduce computational and communication overhead, Miao et al. [11] proposed an innovative lightweight privacy-preserving truth discovery algorithm building upon the previous scheme. This scheme introduces two non-colluding cloud servers, which process the data from data sources in the ciphertext domain using the Paillier homomorphic encryption algorithm and complete the computation of truths through the interaction between the two servers. In 2019, Xu et al. [10] designed a new lightweight truth discovery scheme in combination with the Shamir secret sharing technique and multi-user key agreement to protect users’ sensitive information while realizing that the cloud servers and users are anti-collusion. In 2022, Gao et al. [19] proposed a location privacy-preserving truth discovery scheme, which similarly employs Paillier homomorphic encryption to safeguard users’ location privacy. In the same year, Sun et al. [20] introduced a contract-based personalized privacy-preserving scheme to perturb the aggregated results, allowing users to allocate privacy budgets for their data independently. Compared to traditional differential privacy, this approach offers a more flexible and equitable privacy protection mechanism. In 2024, Peng et al. [21] further advanced the field by proposing a scheme that utilizes additive secret sharing (ASS) to protect data privacy. This ASS-based scheme demonstrates superior computational and communication efficiency over previous approaches based on Shamir secret sharing.

**Verifiability MCS scheme.** As mentioned above, most existing research schemes primarily focus on user privacy protection while paying little attention to the critical issue of data validity. Notably, only a minimal number of research schemes have been designed to address both of these vital dimensions simultaneously. In 2019, Zhang et al. [13] proposed a novel scheme based on Paillier homomorphic encryption, one-way hash chains, and super-increasing sequences. This scheme ensures the validity of each user by verifying their identity, thereby preventing fake users from submitting illegal data. However, it fails to detect anomalous data from legitimate users. Duan et al. [12] introduced a blockchain-driven MCS scheme to address the detection of anomalous data from legitimate users. In this scheme, the cloud server employs ZKRP to filter out outliers, thereby ensuring the validity of the data submitted by users. Nonetheless, this approach requires verifying each data value individually, resulting in low verification efficiency and an inability to defend against the server’s malicious tampering of aggregated results.

Existing schemes exhibit the following limitations: Firstly, the verification process in these schemes entirely relies on the server’s validation of client proofs, making them vulnerable to attacks from malicious servers. Secondly, current schemes based on zero-knowledge proofs struggle to prevent malicious clients from deceiving the server with forged proofs. For instance, a client might generate a valid range proof using correct data but submit invalid data to the server, bypassing the verification mechanism. Thirdly, the existing schemes employ a one-by-one verification mechanism, leading to low verification efficiency and making them unsuitable for large-scale system requirements. Lastly, these schemes lack comprehensive transparency verification throughout the entire truth discovery process of MCS systems. Ideally, a trusted third party should be able to verify every stage of the system, not just the detection of anomalous data, thereby ensuring the overall trustworthiness and fairness of the system.

Our proposed TP-MCS scheme effectively addresses the issues above, as demonstrated in the following three aspects: Firstly, the scheme innovatively integrates ZKP with a Merkle tree structure to establish a dual-verification mechanism. This mechanism not only enables each client to independently verify the integrity of their data during the server’s algorithm execution but also leverages the efficient verification properties of the Merkle commitment tree, allowing a trusted third party to quickly confirm data integrity and consistency by verifying the root node. This collaborative verification mechanism between clients and the trusted third party effectively ensures the integrity and tamper-resistance of the server’s aggregated results. Secondly, the scheme innovatively adapts the truth discovery framework by reorganizing and integrating components, enabling the trusted third party to effectively oversee the entire truth discovery process in the MCS system through zero-knowledge range proof and zero-knowledge inner-product argument. Lastly, the scheme employs an encryption mechanism based on Pedersen commitments, whose hiding properties fully meet the requirements for data privacy protection. This system’s design advances research in federated learning regarding privacy protection and defense against malicious clients and servers, providing a more reliable solution for data processing and analysis in practical application scenarios.

## 3. Preliminaries

In this section, we describe truth discovery, cryptographic technology, Merkle tree, and Merkle commitment tree, which are the foundations of the proposed scheme.

### 3.1. Truth Discovery

In conventional MCS systems, the task publisher first submits the sensing task Task to a cloud server platform, which then recruits suitable participants based on task requirements to collect high-quality sensing data. However, the contribution of sensing data collected in MCS systems is usually different, as the reliability of sensing data provided by different users may differ due to factors such as device variations. Previous research works have introduced the concept of truth discovery to obtain accurate information. Specifically, truth discovery always starts from estimating the reliability $w_{k}$ of each user. Then, it integrates the weights $w_{k}$ of different users for the same object *m* and the sensing data $x_{m}^{k}$ to estimate its groundtruth further. Based on a series of studies in this field [18,22,23], we summarize the general procedure algorithm as follows:

**Weight update.** In this step, the calculation formula for the weight $w_{k}$ of each user k∈[K] is determined based on the given ground truth ${\left\{x_{m}^{*}\right\}}_{m\in [M]}$ of each object, as follows:

(1) $$w_{k}=\log\left(\frac{\sum_{k\in [K]}\sum_{m\in [M]}d\left(x_{m}^{k},x_{m}^{*}\right)}{\sum_{m\in [M]}d\left(x_{m}^{k},x_{m}^{*}\right)}\right)$$

This function assesses the disparity between the data provided by users and the ground truth, assigning higher weights to users whose data are closer to the ground truth. For continuous functions, the construction of the distance function is $s_{k}=d\left(x_{m}^{k},x_{m}^{*}\right)={\left(x_{m}^{k}-x_{m}^{*}\right)}^{2}$, and for categorical data, we use the vector $x_{m}^{k}=\left(0,\cdots ,1^{\left(q\text{-th}\right)},\cdots ,0\right)$ to represent that the q-th option is selected by user *k* for the object *m*. The distance function can be represented as $s_{k}=d\left(x_{m}^{k},x_{m}^{*}\right)={\left(x_{m}^{k}-x_{m}^{*}\right)}^{T}\left(x_{m}^{k}-x_{m}^{*}\right)$. Therefore, for all objects of user *k*, the distance is represented as $s_{k}=\sum_{m\in [M]}d\left(x_{m}^{k},x_{m}^{*}\right)$.

**Truth update.** In this step, based on each user’s weight $w_{k}$ and sensing data $x_{m}^{k}$, we derive the ground truth for each object *m* as follows:

(2) $$x_{m}^{*}=\frac{\sum_{k\in [K]}w_{k}\cdot x_{m}^{k}}{\sum_{k\in [K]}w_{k}}$$

By employing the rule, users with a higher weight $w_{k}$ contribute more significantly to the result of the ground truth $x_{m}^{*}$. This mechanism ensures that $x_{m}^{*}$ tends to favor higher reliability.

### 3.2. Cryptographic Technology

**Commitment scheme.** A commitment scheme [24] Com consists of two algorithms (Com.Setup, Com.Commit). The setup algorithm $\mathrm{Com}.\mathrm{Setup}(1^{\lambda })$ takes the security parameter $1^{\lambda }$ as input and outputs a commitment parameter $p_{c}$. The commitment algorithm $\mathrm{Com}.\mathrm{Commit}(p_{c},x,r)$ takes $p_{c}$, a value *x*, and a randomness *r* as input and outputs a commitment *c*. Finally, the scheme is (additively) homomorphic for all $x_{1}$, $x_{2}\in M_{c}$ and $r_{1}$, $r_{2}\in R_{c}$, and the following exists:

(3) $$\begin{array}{cc} \; & \mathrm{Com}.\mathrm{Commit}\left(p_{c},x_{0},r_{0}\right)+\mathrm{Com}.\mathrm{Commit}\left(p_{c},x_{1},r_{1}\right)\\ \; & =\mathrm{Com}.\mathrm{Commit}(p_{c},x_{0}+x_{1},r_{0}+r_{1}) \end{array}$$

**Non-interactive zero-knowledge proof scheme.** A non-interactive zero-knowledge proof scheme [25] consists of three algorithms (NIZK.Setup, NIZK.Prove, NIZK.Verify). The setup algorithm $\mathrm{NIZK}.\mathrm{Setup}(1^{\lambda })$ takes the security parameter $1^{\lambda }$ as input and outputs a system parameter $p_{zk}$. The prove algorithm $\mathrm{NIZK}.\mathrm{Prove}(p_{zk},sm,w)$ takes $p_{zk}$ and a pair (sm,w) as input and outputs a proof π. The verify algorithm $\mathrm{NIZK}.\mathrm{Verify}(p_{zk},sm,\pi )$ takes $p_{zk}$, a statement sm, and a proof π, and outputs TRUE or FALSE.

**Pseudo-random functions.** The function consists of two algorithms (PRF.KGen,PRF.Eval) [26]. $\mathrm{PRF}.\mathrm{KGen}(1^{\lambda })$ takes the security parameter $1^{\lambda }$ as input and outputs a random key *k*. PRF.Eval(k,x) takes *k* and a message *x* as input and outputs a pseudo-random number *r*.

**Zero-knowledge range proof.** By utilizing the definition of the range proof, we can generate proof that enables the opening of a commitment $c_{r}$, thereby committing to a value within a specific range. To achieve this objective, we establish a set with total ordering on the message space $M_{c}$ and define the range proof for the relationship $R_{r}$ [27].

(4) $$\begin{array}{cc} \; & R_{r}:\left(p_{cr},\left(c_{r},R\right),\left(x,r\right)\right)\in R_{r}\\ \; & ⟷c_{r}=\mathrm{Com}.\mathrm{Commit}\left(p_{cr},x,r\right)\wedge x\in \left[0,R\right] \end{array}$$

**Zero-knowledge inner-product argument.** In the inner-product argument, the prover utilizes the discrete logarithm assumption and employs group exponentiation techniques to conceal the specific values of vectors a and b. Through a series of computations, the prover convinces the verifier that the inner product of vectors a and b equals a common scalar c. The inner-product argument is an efficient proof system for the relation $R_{i}$ [28].

(5) $$\begin{array}{cc} \; & R_{i}:\left(p_{ci},\left(c_{i},c\right),\left(\langle a,b\rangle ,r\right)\right)\in R_{i}\\ \; & ⟷c_{i}=\mathrm{Com}.\mathrm{Commit}\left(p_{ci},\langle a,b\rangle ,r\right)\wedge c=\langle a,b\rangle \end{array}$$

### 3.3. Merkle Tree

The Merkle tree [29] is a unique binary tree type that facilitates quick data verification and retrieval. In a Merkle tree, each data block stores the hash value $h_{j}$ corresponding to node *j*. For intermediate (non-leaf) node *v*, the value is computed as follows: $h_{v}=H(h_{left(v)}\left|\right|h_{right(v)})$, where *H* denotes the cryptographic hash function, left(v) and right(v) represent the left and right child nodes of *v*, respectively. If a child node does not exist, its hash value defaults to 0. Through pairwise hashing and iterative aggregation layers by layers, this structure generates a unique root hash $root_{h}$, which is the digest of the entire data set. This mechanism guarantees data integrity: any modification to the original data alters the hash values along the corresponding path, inevitably changing the root hash. Consequently, the Merkle tree enables efficient tamper detection.

### 3.4. Merkle Commitment Tree

**Figure 1:**
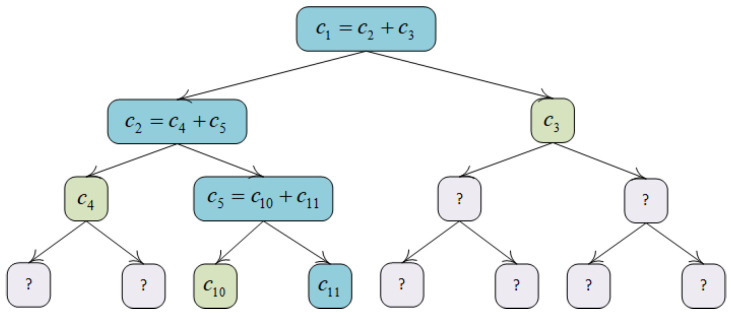
Merkle commitment tree.

In TP-MCS, we introduce a Merkle commitment tree [30] structure with additively homomorphic properties to enable the TA to verify zero-knowledge range proofs efficiently. Specifically, we consider using commitment values to fill the leaf nodes, assuming that the commitment stored in the *j*-th leaf node is $c_{j}=\mathrm{Com}.\mathrm{Commit}\left(p_{c},x_{j},r_{j}\right)$. Based on the additive homomorphism property of the commitment scheme, the commitment of each internal node *v* is the concatenation of the commitment values of its left and right child nodes, i.e., $c_{v}=c_{left(v)}+c_{right(v)}$. (See Figure 1) In this way, the root node value $root_{c}$ contains information from all the sub-nodes in the Merkle commitment tree. The prover can construct an inclusion proof about the target data item *j* by providing the root node value $root_{c}$, the commitment of the j-*th* leaf node, and the commitments of a series of intermediate nodes on the path from that leaf node to the root node. The verifier can confirm whether the target data item *j* is contained in the tree by calculating the commitments layer by layer and comparing the computed root commitment with the root commitment provided by the prover. This process verifies the integrity and consistency of the data.

## 4. Problem Formulation

We first introduce the notations used in the scheme and describe the system model. Next, we present the threat model and design goals.

### 4.1. Notations

In this work, we use the notation *K* to denote $\left\{1,2,\cdots ,K\right\}$ for K∈N. At the same time, we denote the observation entities of the sensing tasks as objects, each user’s reliability is marked as weight, and the truth of each object is represented as groundtruth. A summary of the other notations used in this paper can be found in Table 1.

### 4.2. Design Goals

- *Transparency:* For the entire process of the truth discovery, TA can verify the correctness of each step. In other words, SP cannot convince TA to accept incorrect, incomplete, or manipulated data that lead to erroneous results.
- *Privacy:* Users’ privacy data will remain undisclosed to other users, ensuring their confidentiality throughout information transmission, storage, and processing.
- *Verifiability:* SP cannot tamper with data without detection, as the TA and users can verify the received results for tampering through query requests. This capability ensures the consistency and trustworthiness of information.
- *Efficiency:* Computational costs and communication overhead should be optimal while supporting many users.

### 4.3. System Model

**Figure 2:**
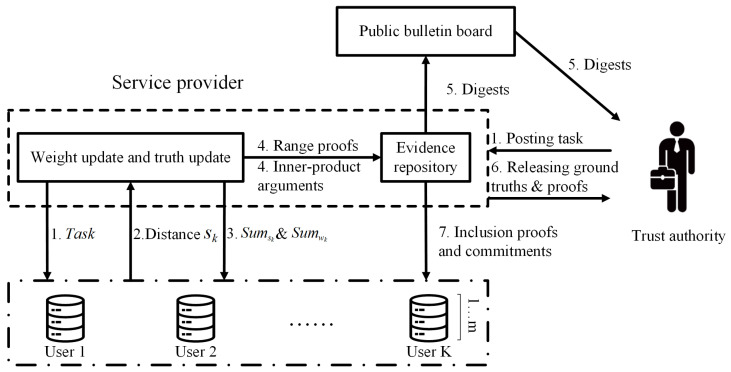
System model of TP-MCS.

We describe our system model in Figure 2, which consists of four main entities: trust authority, service provider, users, and public bulletin board. The definitions of related entities are provided below.

**Trust authority (TA):** TA motivates users to participate in sensing tasks by requesting through SP. Once operational, TA can efficiently verify the correctness of truth discovery.

**Service provider (SP):** SP can collaborate with users to achieve high-quality truth discovery and generate proofs through ZKP technology.

**Users:** Each user *k* collects sensing data through mobile devices and participates in partial verification processes.

**Public bulletin board (PBB):** SP regularly publishes digests to an immutable PBB [30], such as utilizing a public blockchain. Once information is posted on the PBB, it becomes tamper-proof, ensuring the security and traceability of the information.

### 4.4. Threat Model

We consider the TA completely trustworthy, as it does not collude with any participating parties (users or SP), just like in previous MCS systems [31,32,33]. First of all, we also consider two types of threats. One is an adversarial server, which attempts to manipulate data or algorithm execution to produce false aggregation. The other includes semi-adversarial and adversarial users. Semi-adversarial users are honest but curious. They faithfully follow protocols but attempt to learn other users’ private information. Adversarial users may submit anomalous data or deviate from protocol execution.

Then, we assume that a limited set of *f* semi-honest users may collude with SP in a potential attack. In this case, the remaining K−f values will be decrypted only if K−f<2. In other words, at least K−f≥2 honest clients can defend against this attack.

Next, we assume all adversaries are probabilistic polynomial-time (PPT) entities. Under this assumption, they can only break the security of cryptographic primitives—such as discrete logarithm-based commitment schemes and zero-knowledge proofs—with a negligible probability in polynomial time.

Finally, we assume the existence of a PBB, such as a blockchain, which ensures that the stored messages are immutable.

## 5. TP-MCS

In this section, we detail the main steps in designing TP-MCS. Our TP-MCS focuses on two main aspects: (1) We modify the algorithm to reduce verification complexity, making it more efficient and feasible. (2) We develop two protocols: the Merkle commitment tree range proof protocol (MTRP) and the inner-product argument weighted aggregation protocol (IAWAP). These protocols ensure that TA and users can cooperate to supervise each computational step, protecting data privacy.

### 5.1. Truth Discovery Mechanism

The original truth discovery mechanism includes division operations, as shown in Equation (2). In ZKP, division operations typically require calculating inverses, thereby increasing computational overhead. Generally, we strive to prevent the need to verify division operations whenever possible. For ease of verification, we adjust the weight update formula as follows:

(6) $$u_{k}=\log\left(\frac{\sum_{k\in [K]}\sum_{m\in [M]}d\left(x_{m}^{k},x_{m}^{*}\right)}{\sum_{m\in [M]}d\left(x_{m}^{k},x_{m}^{*}\right)}\right)$$

(7) $$w_{k}=\frac{u_{k}}{\sum_{k\in [K]}u_{k}}$$

Then, the truth update formula can be modified as follows:

(8) $$x_{m}^{*}=\sum_{k\in [K]}w_{k}\cdot x_{m}^{k}$$

We can treat Equations (6) and (7) as a black box function f(·). Users directly use f(·) to compute their private $w_{k}$, allowing subsequent matching verification with the commitments declared by SP, thereby avoiding the original division of the plains in Equation (8).

In addition, when processing TP-MCS parameters, we observe that ZKP arithmetic circuits exclusively support integer operations. This limitation arises because ZKP operates over finite fields as a cryptographic construct. Consequently, floating-point numbers must be mapped to the integer domain. To address this, we adopt a fixed-point quantization approach inspired by prior work [34]: defining a scaling factor $L=2^{16}$ and converting a floating-point value $x^{\prime }$ to an integer *x* via $x^{\prime }=\left\lfloor x\cdot L\right\rfloor$. Notably, this will inevitably introduce truncation errors. Therefore, we briefly analyze the error bound as follows: We define the quantization error as $\epsilon =x\cdot L-\left\lfloor x\cdot L\right\rfloor \in \left[0,1\right)$. Since the converted integer value is x’, its corresponding restored value is $\hat{x}=x’/L$. Thus, the absolute error satisfies $\left|x-\hat{x}\right|=\frac{\epsilon }{L}<\frac{1}{L}$. Consequently, when $L=2^{16}$, the error bound is $\left[0,2^{-16}\right)$, which meets the precision requirements of most practical application scenarios. Of course, we can achieve finer quantization for application scenarios requiring higher precision by dynamically adjusting the value of *L*.

Next, we will combine Figure 2 to explain the steps involved in the truth discovery of our scheme. For ease of description, we still represent the transformed data as *x*.

**Step 1. TA $\overset{Task}{\to }$ SP**: TA posts the sensing task to SP, providing information about the task objectives, specific requirements, and other details.

**Step 2. SP $\overset{Task}{\to }$ Users**: SP recruits *K* users to complete the sensing task.

**Step 3. Users $\overset{s_{k}}{\to }$ SP**: Each user *k* computes $s_{k}=\sum_{m\in [M]}d\left(x_{m}^{k},x_{m}^{*}\right)$ of the difference between the sensing data $x_{m}^{k}$ and ground truth $x_{m}^{*}$. Subsequently, users transmit $s_{k}$ to SP.

**Step 4. SP $\overset{Evi^{\prime }}{\to }$ PBB**: SP calculates $Sum_{s_{k}}=\sum_{k\in [K]}s_{k}$. We consider that sensing data beyond the specified range will affect the reliability of the ground truth. Therefore, we assume the sensing data are limited to a specific range $x_{m}\in \left[0,\delta \right]$. Since $s_{k}$ is calculated based on the difference between each user’s sensing data and the ground truth $x_{m}^{*}$, which is publicly available, we assume $s_{k}\in \left[0,\delta_{dist}\right],\delta_{dist}={\left(\delta -x_{m}^{*}\right)}^{2}$, and $Sum_{s_{k}}\in \left[\delta_{dist},\gamma_{dist}\right]$, where $\gamma_{dist}=\delta_{dist}\cdot K-1$. Here, we establish a universal and efficient verification protocol, MTRP, to exclude abnormal sensing data and verify the correctness of the aggregation process. SP executes MTRP to generate evidence $Evi^{\prime }$, i.e., some commitments and proofs about range proofs, and posts it on the PBB. This evidence is used for subsequent verification.

**Step 5. SP $\overset{Sum_{s_{k}},Sum_{w_{k}}}{\to }$ Users:** SP calculates the weights of all users through the function f(·) for weight updating, and the results, $Sum_{s_{k}}$ and $Sum_{w_{k}}$, are sent to the users to update their weights.

Step 6. SP $\overset{Evi^{\prime \prime },{\left\{x_{m}^{*}\right\}}_{m\in [M]}}{\to }$ PBB: SP performs weighted aggregation to complete the truth update. The IAWAP protocol is set to verify the correctness of the execution of Equation (8) based on inner-product argument, preventing malicious behavior by SP. Next, SP takes the evidence $Evi^{\prime \prime }$ and ${\left\{x_{m}^{*}\right\}}_{m\in [M]}$ to PBB.

**Step 7. SP $\overset{c_{k},c_{i},\pi_{k}^{\prime }}{\to }$ Users:** SP sends the commitments and the inclusion proof to each user.

Step 8. SP $\overset{{\left\{x_{m}^{*}\right\}}_{m\in [M]}}{\to }$ TA: SP sends the ground truths to TA.

**Step 9.** TA: TA obtains the digests from the PBB at any time and verifies the evidence $Evi^{\prime }$ and $Evi^{\prime \prime }$ to ensure the correctness of the truth discovery process.

**Step 10. Users:** Each user *k* verifies the consistency of the commitments by computing the commitments based on their private data and comparing them with the commitments declared by SP. In addition, users verify the validity of the inclusion proof provided by the SP by reconstructing the root commitment of the Merkle commitment tree.

### 5.2. MTRP

We describe the specific construction of MTRP in this section. We use commitment schemes and zero-knowledge range proofs to verify the integrity of data aggregation results without compromising user data. Our verification idea is that SP publishes the commitments and proofs on the PBB. Since the PBB is immutable, SP cannot submit the commitments of the original data to the users while simultaneously using the commitments of abnormal data for aggregation. Users execute queries using their private data to verify the consistency of the commitments of the nodes. At the same time, users verify the inclusion proof to prove that their data are included in the tree. TA excludes abnormal sensing data and verifies the correctness of the aggregation process through $root_{c}$, proofs and the commitments of the leaf nodes, thereby maintaining the system’s robustness. The MTRP consists of the four algorithms as follows:

- **$\left(p_{tpr},k_{r}\right)\leftarrow \mathrm{Initialize}\left(1^{\lambda }\right)$**: run by SP. The algorithm initializes the secret key $k_{r}:=\mathrm{PRF}.\mathrm{KGen}\left(1^{\lambda }\right)$ and initializes the commitment and non-interactive zero-knowledge parameter as $p_{cr}$←$\mathrm{com}.\mathrm{Setup}(1^{\lambda })$ and $p_{zkr}\leftarrow \mathrm{NIZK}.\mathrm{Setup}\left(1^{\lambda }\right)$. SP publishes the system parameter $p_{tpr}=\left(p_{cr},p_{zkr},\delta_{dist},\gamma_{dist}\right)$ and keeps the $k_{r}$ private.
- **${\left(r_{k}\right)}_{k\in [K]}\leftarrow \mathrm{SecretGen}\left(p_{tpr},k_{r},k,t\right)$**: run by SP. The algorithm computes the randomness $r_{k}:=\mathrm{PRF}.\mathrm{Eval}(k_{r},k||t)$ for each user *k*, where *t* is the number of iterations.
- **$Evi^{\prime }\leftarrow \mathrm{EviGen}\left(p_{tpr},{\left(s_{k},r_{k}\right)}_{k\in [K]}\right)$**: run by SP. The algorithm computes the commitment of each leaf node $c_{k}\leftarrow \mathrm{COM}.\mathrm{Commit}\left(p_{cr},s_{k},r_{k}\right)$ and the proof $\pi_{k}\leftarrow \mathrm{NIZK}.\mathrm{Prove}\left(p_{zkr},sm,w\right)$, for the statement $sm=(c_{k},\delta_{dist})$ and the witness $w=(s_{k},r_{k})$. Then, SP computes the commitment of $Sum_{s_{k}}$ as $c^{*}\leftarrow \mathrm{COM}.\mathrm{Commit}\left(p_{cr},Sum_{s_{k}},r^{*}\right)$, and the proof $\pi^{*}\leftarrow \mathrm{NIZK}.\mathrm{Prove}\left(p_{zkr},sm,w\right)$, for $sm=(c^{*},\gamma_{dist})$ and $w=(Sum_{s_{k}},r^{*})$, where $r^{*}$ = $\sum_{k\in [K]}r_{k}$. In addition, the server generates the inclusion proofs $\pi_{k}^{\prime }$ for each user. In summary, SP shares the evidence $Evi^{\prime }$ = $\left(c^{*},\pi^{*},{\left(c_{k},\pi_{k}\right)}_{k\in [K]},{\left(\pi_{k}^{\prime }\right)}_{k\in [K]}\right)$ to TA and all users. Subsequently, SP publishes a hash $h^{\prime }=Hash\left(Evi^{\prime }\right)$ on the public PBB.
- **$\left\{0,1\right\}$←$\mathrm{VerifyEvidence}(p_{tpr},{\left(s_{k},r_{k}\right)}_{k\in [K]},Evi^{\prime })$**: run by TA and users. This verification algorithm returns TRUE if four subroutines hold.
  1. $\left\{0,1\right\}\leftarrow \mathrm{VerifyConsistency}\left(Evi^{\prime },h^{\prime }\right)$: TA computes ${\bar{h}}^{\prime }$ = $Hash(Evi^{\prime })$. If ${\bar{h}}^{\prime }$ = $h^{\prime }$, then it outputs TRUE; otherwise, it outputs FALSE.
  2. $\left\{0,1\right\}$←$\mathrm{VerifyCommitment}(p_{cr},s_{k},r_{k},c_{k})$: each user verifies that if ${\bar{c}}_{k}$:=$\mathrm{COM}.\mathrm{Commit}(p_{cr},s_{k},r_{k})$, calculated by each user’s private value $s_{k}$, matches the leaf node promised by SP, i.e., ${\bar{c}}_{k}=c_{k}$, then it outputs TRUE; otherwise, it outputs FALSE.
  3. $\left\{0,1\right\}\leftarrow \mathrm{VerifyInclusionProof}\left(p_{cr},\pi_{k}^{\prime }\right)$: each user verifies the validity of the inclusion proof; i.e., the commitment value of each intermediate node *v* belonging to the proof path is indeed calculated from the commitments of *v*’s children. Then, it outputs TRUE if the proof is valid; otherwise, it outputs FALSE.
  4. $\left\{0,1\right\}\leftarrow \mathrm{VerifySum}\left(p_{zkr},\pi^{*},c^{*},{\left(c_{k}\right)}_{k\in [K]}\right)$: TA computes the root commitment $\bar{c}$ based on ${\left(c_{k}\right)}_{k\in [K]}$. TA verifies whether these three conditions are met: $\bar{c}=c^{*}$, $c^{*}$ matches $\pi^{*}$, and $\mathrm{NIZK}.\mathrm{Verify}(sm,\pi^{*})$ returns true for the statement $sm=(c^{*},\gamma_{dist})$. If all these conditions hold, TA outputs TRUE; otherwise, it outputs FALSE.
  5. $\left\{0,1\right\}\leftarrow \mathrm{VerifyRangeProof}\left(p_{zkr},{\left(c_{k},\pi_{k}\right)}_{k\in [K]}\right)$: TA checks that $c_{k}$ matches the $\pi_{k}$ stored in the leaf nodes and verifies that $\mathrm{NIZK}.\mathrm{Verify}(sm,\pi_{k})$ is true for the statement $sm=(c_{k},\delta_{dist})$. If both conditions hold, it outputs TRUE; otherwise, it outputs FALSE.

### 5.3. IAWAP

Similarly, IAWAP has a verification idea similar to MTRP. Users can calculate commitments based on their private weight $w_{k}$ and sensing data $x_{m}^{k}$. Then, users verify that their commitments are consistent with the commitments declared by the cloud server SP. The third-party TA ensures that SP correctly performs the weighted aggregation step without leaking sensitive information through the inner-product argument.

We use the weight sequence $x=[w_{1},w_{2},\cdots ,w_{K}]$ and the sensing data sequence $y={\left[x_{m}^{1},x_{m}^{2},\cdots ,x_{m}^{K}\right]}_{m\in [M]}$ to calculate the inner product of x and y to obtain an accurate weighted aggregation result.

(9) $$x_{m}^{*}=x\cdot y=\left[w_{1},w_{2},\cdots w_{K}\right]\cdot \left[x_{m}^{1},x_{m}^{2},\cdots x_{m}^{K}\right]$$

For the convenience of subsequent verification, users apply a zero padding strategy to set all data points except for their data values to zero; i.e., the user modifies $x_{w}=w_{k}x_{m}^{k}$ as follows:

(10) $$x_{w}=p_{i}\cdot q_{i}=\left[0,\cdots w_{k},\cdots 0\right]\cdot \left[0,\cdots x_{m}^{k},\cdots 0\right]$$

We define IAWAP as consisting of the four algorithms below.

- $\left(p_{tpi},k_{i}\right)\leftarrow {\mathrm{Initialize}}^{*}\left(1^{\lambda }\right)$: run by SP. The algorithm initializes the secret key $k_{i}$$:=\mathrm{PRF}.\mathrm{KGen}(1^{\lambda })$ and initializes the parameter as $p_{ci}$←$\mathrm{com}.\mathrm{Setup}(1^{\lambda })$ and $p_{zki}$←$\mathrm{NIZK}.\mathrm{Setup}(1^{\lambda })$. Then, SP publishes the system parameter $p_{tpi}=\left(p_{ci},p_{zki},x_{w},x_{m}^{*},K\right)$ and keeps $k_{i}$ private.
- ${\left(r_{i}\right)}_{i\in [K]}\leftarrow {\mathrm{SecretGen}}^{*}\left(p_{tpi},k_{i},i,t\right)$: run by SP. The algorithm computes $r_{i}:=\mathrm{PRF}.\mathrm{Eval}(k_{i},i||t)$ for i∈[K].
- $Evi^{\prime \prime }\leftarrow {\mathrm{EviGen}}^{*}\left(p_{tpi},{\left(\langle p_{i},q_{i}\rangle ,r_{i}\right)}_{i\in [K]},\langle x,y\rangle \right)$: run by SP. The algorithm computes the commitment for each user’s weight and sensing data $c_{i}$:=$\mathrm{COM}.\mathrm{Commit}(p_{ci},\langle p_{i},q_{i}\rangle ,r_{i})$ and for the summed weighted aggregation result ${\hat{c}}^{*}:=\mathrm{COM}.\mathrm{Commit}\left(p_{ci},\langle x,y\rangle ,{\hat{r}}^{*}\right)$, where ${\hat{r}}^{*}=\sum_{i\in [K]}r_{i}$. Then, SP generates the proof ${\hat{\pi }}^{*}\leftarrow \mathrm{NIZK}.\mathrm{Prove}\left(p_{zki},sm,w\right)$, for $sm=({\hat{c}}^{*},x_{m}^{*})$ and $w=(\langle x,y\rangle ,{\hat{r}}^{*})$. In summary, SP shares the evidence $Evi^{\prime \prime }=\left({\left(c_{i}\right)}_{i\in [K]},{\hat{c}}^{*},{\hat{\pi }}^{*}\right)$ to TA and users and sends $h^{\prime \prime }=Hash\left(Evi^{\prime \prime }\right)$ to the public PBB.
- $\left\{0,1\right\}\leftarrow {\mathrm{VerifyEvidence}}^{*}\left(p_{tpi},{\left(\langle p_{i},q_{i}\rangle ,r_{i}\right)}_{i\in [K]},Evi^{\prime \prime }\right)$: run by TA and users. If all three subroutines included in the algorithm pass verification, return TRUE.
  1. $\left\{0,1\right\}\leftarrow {\mathrm{VerifyConsistency}}^{*}\left(Evi^{\prime \prime },h^{\prime \prime }\right)$: TA computes ${\bar{h}}^{\prime \prime }$ = $Hash(Evi^{\prime \prime })$. If ${\bar{h}}^{\prime \prime }$ = $h^{\prime \prime }$, then it outputs TRUE; otherwise, it outputs FALSE.
  2. $\left\{0,1\right\}\leftarrow {\mathrm{VerifyCommitment}}^{*}\left(p_{ci},\langle p_{i},q_{i}\rangle ,r_{i},c_{i}\right)$: users compute ${\bar{c}}_{i}:=\mathrm{COM}.\mathrm{Commit}\left(p_{ci},\langle p_{i},q_{i}\rangle ,r_{i}\right)$, calculated by each user’s private value $w_{k}$ and $x_{m}^{k}$. If ${\bar{c}}_{i}=c_{i}$, then it outputs TRUE; otherwise, it outputs FALSE.
  3. $\left\{0,1\right\}\leftarrow \mathrm{VerifyAgg}\left(p_{zki},{\hat{\pi }}^{*},{\hat{c}}^{*},{\left(c_{i}\right)}_{i\in [K]}\right)$: TA computes ${\bar{c}}^{*}=\sum_{i\in [K]}c_{i}$ and verifies the following conditions: ${\bar{c}}^{*}={\hat{c}}^{*}$, ${\hat{c}}^{*}$ matches ${\hat{\pi }}^{*}$, and $\mathrm{NIZK}.\mathrm{Verify}(sm,{\hat{\pi }}^{*})$ returns true for $sm=({\hat{c}}^{*},x_{m}^{*})$. If the conditions hold, TA outputs TRUE; otherwise, it outputs FALSE.

## 6. Security Analysis

In this section, we focus on TP-MCS’s advantages in terms of transparency and privacy by analyzing the security attributes implemented by the scheme and demonstrating its ability to effectively defend against the security threats described in Section 4.3. Specifically, the combination of transparency, which ensures the public verifiability of system operations, and privacy, which guarantees the confidentiality of user data, enables TP-MCS to comprehensively address various security challenges.

Semi-honest users faithfully follow the protocol but try to learn private information about other users. They may try to disguise themselves as honest users to obtain sensitive information, so we propose Theorem 1: Privacy. Next, we will proceed to prove this theorem.

**Theorem** **1** (Privacy)**.** *If the commitment scheme COM is additively homomorphic, satisfies the hiding property, and adopts an NIZK with zero-knowledge property, which can ensure that sensitive information is not leaked during verification, then TP-MCS guarantees the privacy of the system.*

**Proof.**  Although semi-honest clients do not actively break protocols, their behavior may still threaten to protect the system’s privacy. Their possible malicious behaviors are mainly manifested in the following two aspects:

- Case 1. Semi-honest user *j* tries to disguise itself as an honest user and commits $s_{k}^{\prime }$ to generate false evidence: Due to the hidden attribute of the commitment, even if the semi-honest user obtains the sum of all the values in the Merkle commitment tree according to the protocol, it is still unable to obtain any specific node information. This is because guessing which iteration cycle these values belong to is challenging, and correctly guessing the value of a random seed $r_{k}$ or $r_{i}$ is nearly impossible.
- Case 2. Semi-honest user *j* tries to masquerade as an honest user and tries to use $w_{k}^{\prime }$ or ${x_{m}^{k}}^{\prime }$ to participate in the truth update in order to generate false evidence: even if the semi-honest user computes the homomorphic aggregation result of $x_{m}^{*}$ and all promises based on $w_{k}^{\prime }$ or ${x_{m}^{k}}^{\prime }$, the semi-honest user obtains the result of the homomorphic aggregation ${\hat{c}}^{*}$, but since the hidden attribute is a unidirectional attribute, the semi-honest user is still unable to derive the original input from ${\left\{c_{i}^{\prime }\right\}}_{i\in [K]\setminus \{j\}}$.
- Case 3. Assume that *f* semi-honest users collude with the SP in an attempt to infer the sensitive information of the remaining K−f honest users. Based on the additive homomorphism of commitments and the reversibility of homomorphic operations, SP can remove the commitments of the *f* semi-honest users (whose private data have already been exposed) from the root commitment. This allows the SP to obtain the combined commitment of the K−f≥2 honest clients, denoted as $\sum_{i\in [K-f]}\left\{c_{i}\right\}$. Since our assumptions satisfy the security bound of K−f≥2, the data of a single honest user are masked by the randomness of other honest users, making their private information non-deconstructible in isolation. Conversely, with K−f=1 as an example, the SP has direct access to the commitment of the only honest user, and it knows the sum of the blind factors to infer the original sensitive information. Therefore, the protocol’s security requires at least two honest users and the hidden attributes of the joint commitment, which are sufficient to defend against the conspiracy attack of the semi-honest user and the SP.

Therefore, our solution, TP-MCS, ensures the privacy of the system. □

In the security threat model, both malicious servers and malicious users have the potential to deviate from the protocol. Specifically, a malicious server might tamper with input data or manipulate the algorithm execution process, generating incorrect aggregated computation results. On the other hand, malicious users could submit abnormal or falsified data to corrupt the aggregation results, undermining the system’s integrity and reliability. The transparency attribute of our proposed solution, TP-MCS, can effectively defend against these threats. Consequently, we have proposed Theorem 2: Transparency. Next, we will proceed to prove this theorem.

**Theorem** **2** (Transparency)**.** *If the commitment scheme COM is additively homomorphic and satisfies the binding property, and NIZK is simulation-extractable, which can verify the integrity of data within a polynomial time T, then TP-MCS guarantees the transparency of the system.*

**Proof.**  Both malicious servers and malicious clients may deviate from the established protocols in an attempt to generate erroneous aggregation results, and their malicious behavior may manifest itself in the following three aspects:

- Case 1. If the malicious server tampers with the $s_{v}$ submitted by the honest client *v*, modifying it to $s_{v}^{\prime }$, the aggregation result becomes ${\tilde{Sum}}_{s_{k}}=\sum_{k\in [K]\setminus \{v\}}s_{k}+{\tilde{s}}_{v}$. One scenario is that, in order to hide the fact of tampering with $s_{v}$, the malicious server submits to the bulletin board the correct leaf node commitment values ${\left\{c_{k}\right\}}_{k\in [K]}$ in order to evade the client’s inspection ${\bar{c}}_{v}=\mathrm{COM}.\mathrm{Commit}\left(p_{cr},s_{v},r_{v}\right)=c_{v}$. In this case, TA performs the computation ${\tilde{c}}^{*}=\mathrm{Com}.\mathrm{Commit}\left(p_{cr},{\tilde{Sum}}_{s_{k}},r^{*}\right)$, which will reject the aggregated result with a probability of 1−negl(κ). In another case, in order to evade the TA’s validation, the malicious server submits the commitments $c_{k\in [K]\setminus \{v\}}$ and ${\tilde{c}}_{v}$ to the bulletin board, which will not be filtered out by the TA performing the computation. However, since the bulletin board is tamper-proof and the commitments have binding properties, the probability that a client will collide with ${\tilde{c}}_{v}=\mathrm{COM}.\mathrm{Commit}\left(p_{cr},s_{v}^{\prime },r_{v}\right)$ in verifying its real commitment $c_{v}$ is almost negligible, and thus it will be detected by an honest client.
- Case 2. If a malicious server tampers with the $w_{u}$ or $x_{m}^{u}$ submitted by an honest client, modifying it to $w_{u}^{\prime }$ or ${x_{m}^{u}}^{\prime }$, the aggregation result becomes ${\tilde{x}}_{m}^{*}=\sum_{i\in [K]\setminus \{u\}}w_{k}\cdot x_{m}^{k}+{w_{u}}^{\prime }\cdot {x_{m}^{u}}^{\prime }$. One scenario is that the server submits the correct leaf node commitment values ${\left\{c_{i}\right\}}_{i}\in \left[K\right]$ to the bulletin board in order to hide the tampering to avoid the client’s inspection. However, in this case, the TA performs the computation ${\tilde{\hat{c}}}^{*}=\mathrm{Com}.\mathrm{Commit}\left(p_{ci},{\tilde{x}}_{m}^{*},{\hat{r}}^{*}\right)$ and will reject the result with a probability of 1−negl(κ). Another scenario is to submit the commitment $c_{i\in [K]\setminus \{u\}}$ and $c_{u}$ to the bulletin board and thus be able to evade the TA’s computation. But since an honest user can generate the correct commitment ${\tilde{c}}_{u}=\mathrm{COM}.\mathrm{Commit}\left(p_{ci},\left(\langle w_{u}^{\prime },{x_{m}^{u}}^{\prime }\rangle \right),r_{u}\right)$ with his own private data, and due to the binding property of the promises, the probability of $c_{u}$ and ${\tilde{c}}_{u}$ colliding is negligible.
- Case 3. If a malicious client submits anomalous data $s_{v}^{\prime }$ such that $s_{v}^{\prime }>\delta_{dist}$, or $w_{u}^{\prime }$ and ${x_{m}^{u}}^{\prime }$, it will result in the server failing to validate the zero-knowledge range proof and zero-knowledge inner-product argument that it generates on this basis. This is because the TA will be able to identify such anomalous behavior by detecting verification failures and verifying the evidence uploaded to the bulletin board by the server.

In summary, the TP-MCS is fully transparent because the TA can verify the correctness of each step throughout the process of truth discovery. □

## 7. Experimentation and Performance Evaluation

To evaluate our proposal, we developed a prototype system utilizing a laptop with an AMD Ryzen 9 5900HX processor and a 3.30 GHz Radeon Graphics card, and experimented with it in an Ubuntu 22.04 environment. We implemented TP-MCS based on the ZKRP library [35], which uses Pedersen commitment with the secp256k1 elliptic curve to construct the commitment scheme. In practice, we employed a baseline solution that does not consider any security factors, i.e., CRH [18], to evaluate the accuracy and convergence of our scheme. We also conducted a performance assessment to demonstrate the feasibility of the scheme.

### 7.1. Accuracy

**Figure 3:**
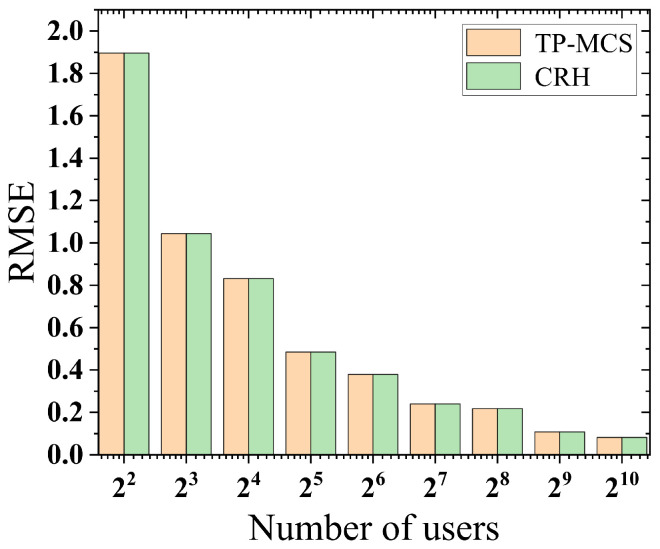
Accuracy evaluation.

Similar to previous research methods [9,11], we employed the root mean squared error (RMSE) to quantify the difference between the estimated ground truths and the actual ground truths. We set the number of objects as 16, the number of iterations as 20, and varied the number of users across $2^{2},2^{3}\cdots ,2^{10}$. We used a random initialization method to generate sensing data for each object, where the range of values for each data point is controlled to be between the minimum and maximum values, and the spacing is limited to five units. Subsequently, we initialized the actual ground truth by calculating the arithmetic mean of these randomly generated data. In Figure 3, we present the measurement results, showing that TP-MCS and CRH have nearly the same estimation accuracy.

**Figure 4:**
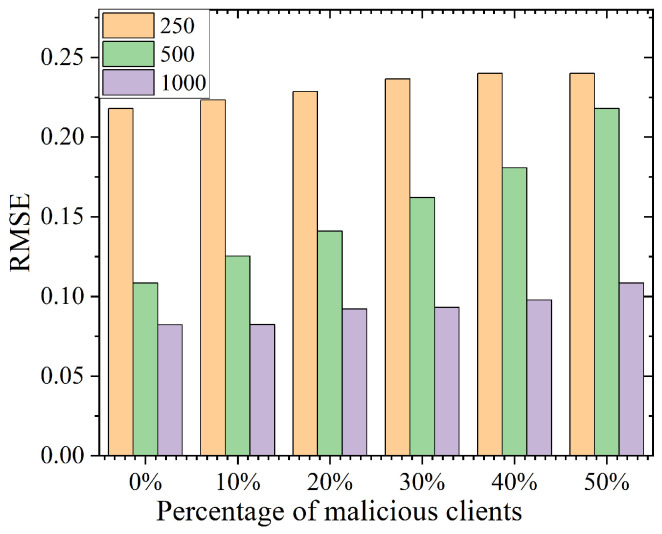
Accuracy evaluation with involvement of malicious users.

Similarly, we set the number of objects as 16 and the number of iterations as 20, and we used the sensing data generation scheme described above. As shown in Figure 4, to evaluate the system’s robustness, we systematically investigated the change in RMSE when the percentage of malicious users was incremented from 10% to 50% at user sizes of 250, 500, and 1000, respectively. The experimental design assumed that malicious users generated data beyond the constraint range, and all unvalidated user data were excluded from the final aggregation process. The experimental results show that in the scenario of 500 clients, the RMSE difference between different malicious user ratios was only about 0.1. When the user scale was expanded to 1000, the RMSE value was stable below 0.1. It fluctuated within 0.02, which indicates that our scheme has a significant anti-interference ability against anomalous data injected by malicious users.

### 7.2. Convergence

**Figure 5:**
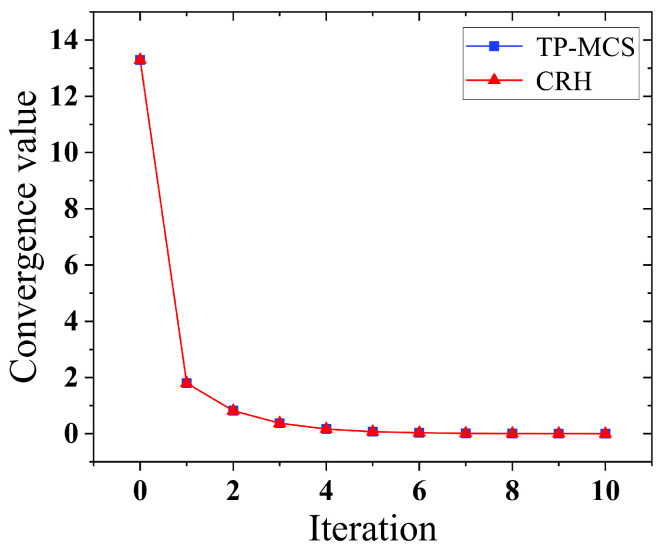
Convergence evaluation.

We further analyzed the convergence of the proposed scheme. We defined $\left|{x_{m}^{*}}^{t}-{x_{m}^{*}}^{t-1}\right|$ to evaluate the difference between the estimated truths in consecutive iterations. The initial value ${x_{m}^{*}}^{t=0}$ was randomly initialized. As shown in Figure 5, TP-MCS exhibits similar convergence capabilities to CRH, rapidly converging within a few iterations.

### 7.3. Performance Evaluation of TP-MCS

**Communication overhead.** To optimize performance, we focus on two main aspects. We first use the Merkle tree to reduce the overhead of range proof. For *K* users, SP generates commitments for 2K−1 nodes and constructs range proofs based on the leaf and root nodes. When TA verifies the range proof of a node in the tree, SP only needs to send the commitments included in the range proof, which means verifying $\log_{2}^{K}$ commitments along the adjacent path and the corresponding $\log_{2}^{K}+1$ additional child node commitments, totaling $2\log_{2}^{K}+1$ commitments, without traversing the entire Merkle tree, significantly improving efficiency. Then, users are often constrained by device resources. Therefore, we discuss blockchain-based protocol extensions to reduce the communication overhead on the user side. We observe that only VerifyCommitment, ${\mathrm{VerifyCommitment}}^{*}$, and VerifyInclusionProof need to be verified by users. Therefore, other verifications can be executed by TA, undoubtedly reducing the users’ cost. TA executes VerifyConsistency, ${\mathrm{VerifyConsistency}}^{*}$, VerifySum, VerifyRangeProof, and VerifyAgg on a blockchain smart contract platform to verify the commitments and proofs generated in our protocol. If these verifications pass, the blockchain will store the relevant messages. Then, each user can directly read the stored commitments and proofs and use their private data to compute ${\bar{c}}_{k}$ and ${\bar{c}}_{i}$ to evaluate the consistency of the commitments without incurring consensus overhead.

We show in Table 2 the performance cost of performing the TP-MCS scheme when using smart contracts. We use the message size to represent the broadband cost in the network. We denote the size of commitments and proofs in MTRP as ${M_{c}}_{r}$, ${M_{\pi }}_{r}$, and IAWAP as ${M_{c}}_{i}$, ${M_{\pi }}_{i}$. $M_{t}$ represents the message stored in the blockchain, waiting for read requests. We find in Table 2 that unloading messages from the blockchain not only reduces communication overhead between each entity, but also allows for the public verification of the authenticity and integrity of the information submitted by SP, due to the immutable nature of the commitments and proofs stored on the blockchain, thereby enhancing the system’s transparency and credibility.

**Computation overhead.** Unlike existing research that primarily focuses on privacy protection mechanisms, the scheme proposed in this study innovatively introduces the MTRP protocol based on ZKRP and the Merkle tree and the IAWAP protocol based on inner-product arguments. This scheme ensures privacy and, for the first time, achieves the function of transparent and public verification, effectively addressing the shortcomings in the verifiability of existing solutions. Given this scheme’s innovative breakthrough in functionality, it is imperative to evaluate its performance systematically. To this end, this study designs a set of comparative experiments aimed at deeply exploring the impact of changes in user scale (from $2^{1}$ to $2^{10}$) and object scale (from $2^{1}$ to $2^{9}$) on system performance, providing important empirical evidence for its application deployment in real-world scenarios.

**Figure 6:**
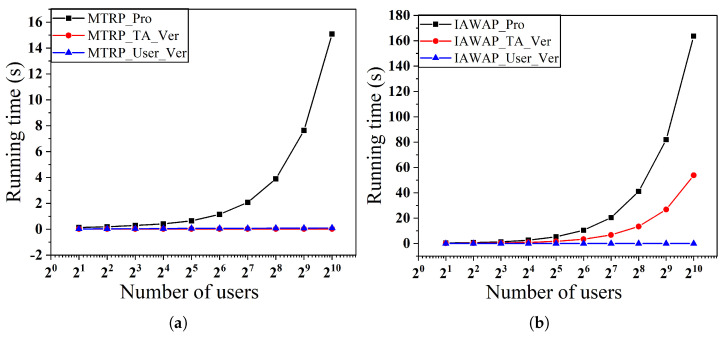
(**a**) Proof of generation and verification time with varying numbers of users of MTRP. (**b**) Proof of generation and verification time with varying numbers of users of IAWAP.

**Figure 7:**
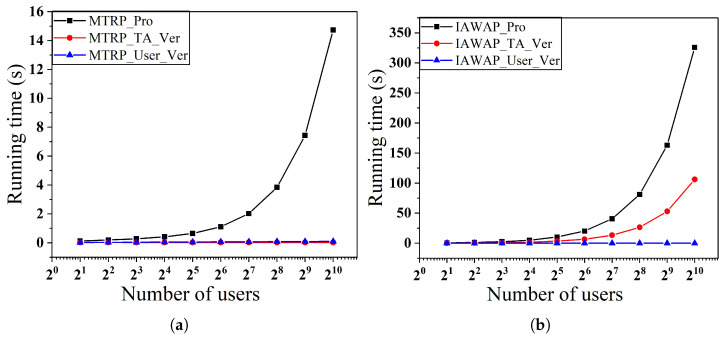
(**a**) Proof of generation and verification time with varying numbers of users of MTRP. (**b**) Proof of generation and verification time with varying numbers of users of IAWAP.

In the first set of experiments, as shown in Figure 6 and Figure 7, we examine the effect of varying the number of users on the time overhead for a fixed number of objects, respectively. Specifically, Figure 6a,b show the performance when the number of objects is fixed at $2^{8}$ and the number of users varies from $2^{1}$ to $2^{10}$, and Figure 7a,b show the corresponding results when the number of objects is fixed at $2^{9}$.

We observe that in the MTRP protocol, the verification time overhead is low and stable, which is due to the introduction of the Merkle tree data structure, allowing the TA to only need to verify the root hash to complete the integrity verification of all the data, thus avoiding the tedious process of verifying all the participant’s data one by one, ultimately achieving a constant-level verification time complexity. In addition, the overhead of generating proofs for the MTRP and IAWAP protocols and the overhead of TA verification proofs in the IAWAP protocol grow linearly with the number of users, which is controllable and acceptable in practice. Also, in both protocols, users only need to validate their data, which gives them inexpensive computational complexity.

**Figure 8:**
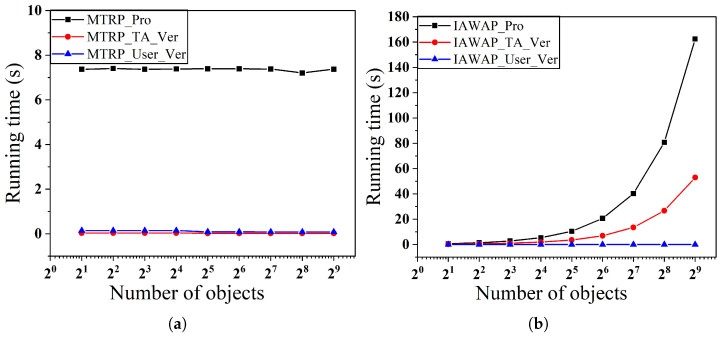
(**a**) Proof of generation and verification time with varying numbers of users of MTRP. (**b**) Proof of generation and verification time with varying numbers of users of IAWAP.

**Figure 9:**
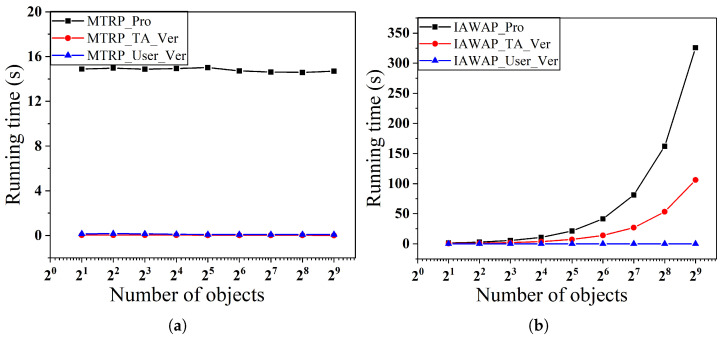
(**a**) Proof of generation and verification time with varying numbers of users of MTRP. (**b**) Proof of generation and verification time with varying numbers of users of IAWAP.

In the second set of experiments, as shown in Figure 8 and Figure 9, we examine the effect of varying the number of objects on the time overhead for a fixed number of users, respectively. Specifically, Figure 8a,b show the performance when the number of users is fixed at $2^{9}$ and the number of objects varies from $2^{1}$ to $2^{9}$, and Figure 9a,b show the corresponding results when the number of users is fixed at $2^{10}$.

In scenarios with a fixed number of users, the proof generation time of the MTRP protocol exhibits notable stability, with its time cost being entirely independent of the number of objects, consistently maintaining a constant level. In contrast, the proof generation time of the IAWAP protocol and the TA verification time remain linearly positively correlated with changes in the number of objects. Meanwhile, the verification time and the MTRP protocol’s user verification time in the IAWAP protocol consistently tend toward zero.

**Figure 10:**
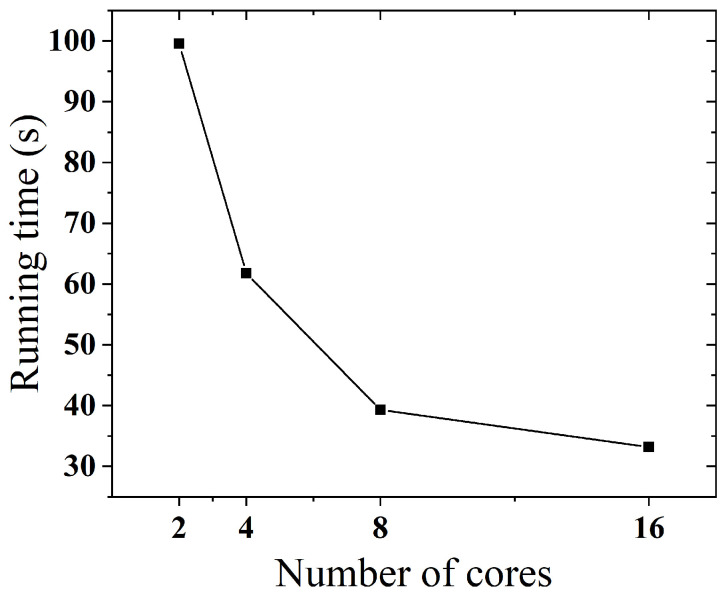
Impact of core count on performance.

Next, we set a fixed value of $2^{10}$ users and 16 objects to study the variation in execution time under different numbers of cores. As shown in Figure 10, we find that TP-MCS can effectively scale with more CPU cores. For example, when the number of cores ${\mathrm{num}}_{core}=16$, the runtime reduces by 66.38 s compared to ${\mathrm{num}}_{core}=2$.

### 7.4. Comparison to Existing Schemes

We compare our scheme with other schemes, including security properties and performance overheads.

**Comparison of properties**. Table 3 compares our scheme with other schemes regarding security attributes, including privacy, transparency, verifiability, and efficiency. The baseline scheme [18] can only identify truthful values in crowd-sensing systems but fails to meet other security attributes, such as user privacy. The works [11,13,19] use Paillier encryption to ensure user privacy, though this approach incurs high overhead. Among these, the work [13] introduced an identity verification mechanism capable of effectively identifying and authenticating malicious users, thereby reliably excluding illegal users from the system. Sun et al. [20] optimized traditional differential privacy schemes by allowing users to customize their privacy budgets, significantly enhancing the flexibility and fairness of the approach. In addition, mbox [21] utilizes additive secret sharing (ASS) to protect data privacy, which is superior in terms of computational efficiency and communication overhead compared to the traditional Shamir secret sharing scheme. In addition, the work [12] combines blockchain, Paillier encryption, and zero-knowledge proof technology to obtain the privacy and verifiability of submitted data, but this scheme is inefficient and non-transparent. In contrast, our scheme combines commitment schemes and zero-knowledge proofs, ensuring operational transparency and verifiability while protecting user privacy. We construct a Merkle commitment tree to improve protocol efficiency further, significantly reducing the protocol’s computational overhead.

**Comparison of overhead.** We assume that there exists a server provider SP, *K* clients, and *M* objects, and that the size of the task is *a*, the size of $x_{m}^{*}$ is *d*, and the size of $s_{k}$ is *n*. From Equations (6)–(8), we derive the size of $Sum_{s_{k}}$ to be Kn, and the size of $Sum_{w_{k}}$ to be logK. We ignore the overhead of TA for this analysis since we do not include the entity in our comparison scheme. TA has performed only one simple verification computation, which has a negligible impact on the asymptotic complexity of the overall scheme. We report our scheme’s computational overhead and communication overhead versus the other schemes on both the server and client sides in Table 4.

*Server computation overhead.* (1) SP generates for each client an inclusion proof $\pi_{k}^{\prime }$ that includes all the commitments on the path from the self-leaf node to the root node. Thus, the total computational complexity of generating inclusion proofs for *K* clients is O(K(logK+logn)). (2) SP generates a range proof for $s_{k}$ for each client. The Bulletproof protocol we employ compresses the proof size by recursively reducing it from linear complexity to a logarithmic level. Specifically, each step of recursive compression splits a vector of length *n* into two vectors of length n/2, gradually reducing the computational size. As a result, the complexity of a range proof for *K* clients is O(Klogn). (3) SP computes the summing operation of $Sum_{s_{k}}$ and $Sum_{w_{k}}$ with a computational complexity of O(K). (4) SP generates the inner-product argument and performs truth discovery. The vector dimensions of x and y are *K*. Then, the computational complexity of performing the inner-product argument protocol is O(logK), and the computational complexity of computing the inner product of vectors is O(K). Thus, the computational complexity is O(K+logK). In summary, the total computational complexity on the server side is O(K(logK+logn)). Compared to other schemes, the work [18] does not introduce any cryptography and has minimal computational overhead. In the previous work [11,13,19], SP has to decrypt the result of Paillier encryption for *K* clients. The work [11] is a dual server, and separate decryption is required between the servers. Assuming that the size of the noise in the scheme Sun et al. [20] is $M_{n}$, the communication overhead of the server will increase by $O(KM_{n})$ compared to the privacy-preserving scheme without privacy protection [18]. In work [21], the computational overhead of the server mainly comes from the random number generation and addition operations in the secret sharing phase and the addition operations in the secret reconstruction phase. Specifically, generating *K* shared values requires K−1 random number generation and one addition operation, while reconstructing the secret requires K−1 addition operations. Therefore, the computational overhead is O(K). Moreover, the work [12] requires not only verifying the zero-knowledge proof but also decrypting Paillier encryption, and thus has the highest computational complexity.

*Server communication overhead.* (1) SP sends the task, $Sum_{s_{k}}$, $Sum_{w_{k}}$, and $x_{m}^{*}$ to *K* clients, and we assume that V=a+d+Kn+logK, so the communication complexity is O(V). (2) From Table 2, we assume that $M_{s}=\left(K+1\right)\left({M_{c}}_{r}+{M_{\pi }}_{r}+{M_{c}}_{i}+{M_{\pi }}_{i}\right)+K\left(2log_{2}^{K}+1\right){M_{c}}_{r}$, so the communication complexity is $O(M_{s})$. Thus, the communication overhead of the server is $O(V+M_{s})$. Assuming that the size of the transmitted Paillier homomorphic encrypted data is $M_{e}$, in general, $M_{e}>>M_{s}$. Thus, the communication complexity of the server is lower than in the works [11,12,13,19], but higher than in the schemes [18,20,21], because the server has to send commitments and proofs.

*Client computation overhead.* (1) The client computes its own $s_{k}$ with a complexity of O(M). (2) The client computes the commitment for verification with a complexity of O(logn). (3) The client verifies the inclusion proof with a complexity of O(logK). Therefore, the total computational complexity for the client is O(M+logn+logK). The client in scheme [18] only needs to compute the value of $s_{k}$, and the client of [20] only needs to perform a noise add operation on the local data, which makes its computational complexity comparable to that of [18]. Thus, they have the lowest computational complexity of all the schemes. At the same time, the client in [11,12,13,19] requires additional Paillier encryption operations. The client in [21] needs to perform element-by-element randomized partitioning and shared computation on data $s_{k}$ of size *n*. Therefore, the TP-MCS client is less efficient than most schemes in terms of computational complexity and allows the client to perform query validation.

*Client communication overhead.* (1) The client sends $s_{k}$ to SP with O(n) communication complexity. (2) The client feeds back an error flag to the PBB when it verifies that the commitment is inconsistent or that the inclusion proof is incorrect, with a communication complexity of $O(M_{u})$, where $M_{u}=\left(2log_{2}^{K}+1\right){M_{c}}_{r}+{M_{c}}_{i}+2M_{t}$. Therefore, the total communication complexity for the client is $O(n+M_{u})$. Compared to other schemes, the client of TP-MCS has higher communication complexity, because the client needs to verify the inclusion proof and commitment consistency through Merkle tree path information to prevent tampering by malicious servers. Such a verification mechanism is not available in other schemes.

## 8. Discussion and Limitations

The TP-MCS system proposed in this paper realizes the public verification of the execution process of the MCS truth discovery algorithm in privacy-preserving scenarios. It effectively filters out malicious clients, thus enhancing the trust of TA in the MCS system. However, the system still has some limitations. First, the use of zero-knowledge proof techniques introduces additional computational overhead. For example, the computational complexity of the IAWAP protocol is positively correlated with the number of users and objects, which can be a performance bottleneck in large-scale data deployment scenarios. In future work, distributed computing schemes such as federated learning can reduce the computational overhead and improve the system’s scalability by training data locally on the user side and aggregating them securely on the server side [36]. Second, the transparent design of the system, especially the validation part, relies on the active participation of users. However, some users may not fully utilize this feature, resulting in a limited practical effect of transparency. To improve user participation, reasonable incentives [37], such as financial rewards or reputation points, can be designed in the future to encourage users to participate actively in the verification process.

In summary, although the TP-MCS system has made significant progress in privacy protection and transparency, it needs to be further optimized regarding computational efficiency and user participation to better adapt to large-scale practical application scenarios.

## 9. Conclusions

In this paper, we creatively designed a transparent and privacy-preserving truth discovery system. The scheme combines commitment schemes and zero-knowledge proofs to guarantee data privacy while allowing the verification of the correctness of data aggregation results. We also designed Merkle commitment trees to reduce computational overhead further. Meanwhile, we performed a security analysis of the scheme and discussed the optimization scheme based on blockchain extension. In addition, we conducted comprehensive experiments and compared our scheme with other state-of-the-art schemes, demonstrating that our scheme outperforms them in terms of accuracy and computational cost.

## Figures and Tables

**Table 1:** Summary of the notations used in this paper.

Symbol	Value	Description
*K*	N	number of users
*M*	N	number of objects
*C*	N	number of time periods per operational cycle
*T*	1	trust authority
Task	/	the sensing task
$x_{m}^{k}$	$\left\{x_{m}^{1},\ldots ,x_{m}^{K}\right\}$	the sensing data of the object *m* for each user k∈K
$x_{m}^{*}$	N	the ground truth of each object m∈M
$s_{k}$	$\left\{s_{1},\ldots ,s_{K}\right\}$	the *k*-th user’s summed distance
$Sum_{s_{k}}$	N	the summed distance of each user k∈K
$w_{k}$	$\left\{w_{1},\ldots ,w_{K}\right\}$	the weight of each user k∈K
δ	N	the threshold of sensing data
$\delta_{dist}$	N	the threshold of $s_{k}$
$\gamma_{dist}$	$\left\{0,\delta_{dist}*K-1\right\}$	the threshold of $Sum_{s_{k}}$
$c_{k}$	$C_{c}$	the commitment of $x_{m}^{k}$
$c^{*}$	$C_{c}$	the sum of $c_{k}$
$r_{k}$	$R_{c}$	random secret of each user k∈K
$\pi_{k}$	/	zero-knowledge range proof of $x_{m}^{k}\in \left[0,\delta_{dist}\right]$
$\pi_{k}^{\prime }$	/	the inclusion proof of each user k∈K
$\pi^{*}$	/	zero-knowledge range proof $Sum_{s_{k}}\in \left[\delta_{dist},\gamma_{dist}\right]$
$c_{i}$	$C_{c}$	commitment of the *i*-th user’s $w_{k}$ and $x_{m}^{k}$
${\hat{c}}^{*}$	$C_{c}$	commitment of the sequence $w_{k}$ and $x_{m}^{k}$ for i∈K
$r_{i}$	$R_{c}$	random secret of user i∈K
${\hat{\pi }}^{*}$	/	inner-product argument of the $w_{k}$ and $x_{m}^{k}$ for i∈K

**Table 2:** The communication overhead of TP-MCS in smart contracts.

Entity	Computation			Bandwidth
	com.Commit	nizk.Prove	nizk.Verify	
SP	3K−1	2K+2	0	$\left(K+1\right)\left({M_{c}}_{r}+{M_{\pi }}_{r}+{M_{c}}_{i}+{M_{\pi }}_{i}\right)+K\left(2log_{2}^{K}+1\right){M_{c}}_{r}$
User	2	0	2	$\left(2log_{2}^{K}+1\right){M_{c}}_{r}+{M_{c}}_{i}+2M_{t}$
TA	0	0	2	${M_{c}}_{r}+{M_{\pi }}_{r}+{M_{c}}_{i}+{M_{\pi }}_{i}+2M_{t}$
PBB	0	0	0	$2\left(K+1\right)M_{t}$

**Table 3:** Comparison of properties.

Scheme	Privacy	Transparency	Verifiability	Efficiency
Li et al. [18]	✗	✗	✗	✓
Miao et al. [11]	✓	✗	✗	✗
Zhang et al. [13]	✓	✗	✓	✗
Duan et al. [12]	✓	✗	✓	✗
Gao et al. [19]	✓	✗	✗	✗
Sun et al. [20]	✓	✗	✗	✓
Peng et al. [21]	✓	✗	✗	✗
TP-MCS	✓	✓	✓	✓

✓ indicates that the property is satisfied. ✗ indicates that the property is not satisfied.

**Table 4:** Performance comparison.

Scheme	Server Overhead		Client Overhead	
	Computation	Communication	Computation	Communication
Li et al. [18]	O(K)	O(V)	O(M)	O(n)
Miao et al. [11]	$O(\left(K+1\right)n^{2})$	$O(V+M_{e})$	$O(M+n^{2})$	O(n)
Zhang et al. [13]	$O(Kn^{2})$	$O(V+M_{e})$	$O(M+n^{2})$	O(n)
Duan et al. [12]	$O(K\left(\log n+n^{2}\right))$	$O(V+M_{e}+K{M_{\pi }}_{r})$	$O(M+n^{2})$	$O(n+{M_{\pi }}_{r})$
Gao et al. [19]	$O(Kn^{2})$	$O(V+M_{e})$	$O(M+n^{2})$	O(n)
Sun et al. [20]	O(K)	$O(V+KM_{n})$	O(M)	$O(n+M_{n})$
Peng et al. [21]	O(Kn)	O(V+Kn)	O(M+n)	O((K−1)n)
TP-MCS	O(K(logK+logn))	$O(V+M_{s})$	O(M+logn+logK)	$O(n+M_{u})$

## Data Availability

All data are contained within the article.
